# Establishment of a predictive model for postpartum hemorrhage in twins: a retrospective study

**DOI:** 10.1186/s12884-023-05933-7

**Published:** 2023-09-07

**Authors:** Sangsang Qi, Xianhu Fu

**Affiliations:** 1 Department of Obstetrics and Gynecology, Ningbo Women and Children’s Hospital, No. 339 Liuting Street, Haishu District, 315012 Ningbo, China; 2 School of Medicine, Ningbo University, Ningbo, China

**Keywords:** Twins, Postpartum hemorrhage, Prediction model

## Abstract

**Objective:**

To explore the risk factors and develop a predictive model for postpartum hemorrhage in twin pregnancies.

**Methods:**

All patients who gave birth at Ningbo Women and Children’s Hospital from January 2018 to August 2022 were recruited. Patients were randomly allocated to a training cohort (n$=$1395) validation cohort (n$=$650) at a 7:3 ratio. In the training cohort, LASSO regression for screening variables and multifactorial logistic regression analysis were performed to identify independent risk factors for postpartum hemorrhage in twin pregnancies. A nomogram was established based on the results of multiple logistic regression analysis. Nomogram performance was quantified using the receiver operating characteristic curve, Hosmer- Lemeshow test and decision curve analysis.

**Results:**

A total of 2045 patients were included in this study. Multifactorial Logistic regression analysis showed maternal age, assisted reproduction, platelet count, fibrinogen level, albumin level, hypertensive disorders of pregnancy, placenta praevia, number of previous cesarean deliveries, number of previous intrauterine manipulation, and neonatal weight were independent risk factors for postpartum hemorrhage in twin births. The area under curve (AUC) for the training cohort was 0.810 [95$\%$ CI (0.781, 0.839)], with a sensitivity of 76.5$\%$, specificity of 71.0$\%$, and positive and negative predictive values of 0.358 and 0.935, respectively, while the AUC for the validation cohort was 0.821 [95$\%$ CI (0.781, 0.860)], with a sensitivity of 80.9$\%$, specificity of 69.49$\%$, and positive predictive value and negative predictive value of 0.426 and 0.929, respectively.

**Conclusion:**

The predictive model can effectively and quantitatively assess the risk of postpartum hemorrhage in twin pregnancies and help clinicians to take personalized preventive measures.

**Supplementary Information:**

The online version contains supplementary material available at 10.1186/s12884-023-05933-7.

## Background

Postpartum hemorrhage (PPH) is a serious complication during labour and delivery, and the incidence of PPH accounts for 3$\%$-5$\%$ of all deliveries and is the leading cause of maternal mortality worldwide [1]. With the development of assisted reproductive technologies, the number of twin pregnancies has increased significantly in recent years. Due to the hyperextension of uterine fibres in twin pregnancies, resulting in poor uterine contraction at the placental attachment site after delivery, the risk of PPH in twin pregnancies is significantly higher than in singleton pregnancies, and severe PPH may endanger a woman’s life [2].

PPH can result in a series of hazards. Some studies [3] have indicated that establishing a predictive early warning system for significant diseases, monitoring pregnant women with high-risk factors and quantifying and scoring these high-risk factors can provide early identification, timely intervention, and treatment and help reduce the occurrence of PPH. The nomogram [4] is a reliable statistical model that provides individualized and highly accurate risk assessment and is now widely used in obstetrics and gynaecology, such as the nomogram for predicting survival after uterine sarcoma [5] and the nomogram for the survival of patients with cervical cancer [6]. In this study, the prediction of the risk of PPH in twin pregnancies was conveniently and visually represented in the form of a nomogram. The objective of this study was to develop a more accurate, stable, and applicable model to predict the risk of PPH in twin pregnancies. We believe that our study can be used to strengthen prenatal management and evaluation as well as provide a reference for the prevention of the risk of PPH in twin pregnancy and appropriate intervention strategies.

## Methods

### Diagnostic criteria for PPH

According to the American College of Obstetricians and Gynecologists (ACOG) 2017 definition, PPH was defined in this study as a cumulative amount of bleeding $\ge$ 1,000 ml within 24 hours after delivery.

### Measurement methods of PPH

For vaginal deliveries, during the third stage of labour, a blood storage dish was inserted under the mother’s buttocks, and a measuring cup was used to determine the blood volume. After the third stage of labour, a nursing pad was placed beneath the buttocks and left in place for 24 hours. The used nursing pad was weighed, and the original weight was subtracted. To calculate the blood volume, the weight differential was multiplied by 1.05. The entire amount of bleeding for the first 24 hours following birth was added to the amount mentioned above.

For cesarean sections, the amniotic fluid was first aspirated after the uterine wall was incised. The pressure suction bottle was replaced, and intraoperative blood was collected. A nursing pad was placed under the mother’s buttocks after she returned to the ward. The amount of bleeding was calculated by the weighing method, and the cumulative amount of blood was added to determine 24-h postpartum bleeding.

### Ethics approval and consent to participate

The study was institutionally approved by the Ethics Committee of Ningbo Women and Children’s Hospital, and informed consent was waived because the study was retrospective.

### Study population

Women with twin pregnancies who delivered at Ningbo Women and Children’s Hospital between January 2018 and August 2022 with a gestational age $\ge$ 28 weeks were included.

#### Inclusion criteria

The inclusion criteria were women with twin pregnancies who gave birth in our institution at a gestational age $\ge$ 28 weeks and had a routine obstetric examination.

#### Exclusion criteria

1. Patients who did not have a standardized obstetric examination and for whom clinical data were not available.
2. Patients with systemic diseases, including multiple malignant tumours, mental illness, clotting issues, severe cardiovascular and liver conditions, haematologic conditions, and serious surgical diseases.

### Variables included in statistics

The study team completed all statistical data by double-checking. The risk factors for postpartum hemorrhage in twin births were established by reading the literature and team discussion and included the following: (1) general information: maternal age, number of miscarriages, gestational weeks at delivery, prepregnancy body mass index (BMI), type of conception, and type of chorionic membrane; (2) prenatal factors: platelet count (PLT), fibrinogen (FIB), and albumin levels (within three days before delivery). gestational diabetes mellitus (GDM), hypertensive disorders of pregnancy (HDP), intrahepatic cholestasis of pregnancy (ICP), immune system disorders and thrombophilia, uterine myoma, prelabour rupture of membranes, polyhydramnios, placental abruption, and placenta praevia; (3) previous factors: number of previous cesarean deliveries, number of previous vaginal deliveries, and history of previous PPH; (4) delivery factors: surgeon’s seniority, intrauterine infection, mode of delivery, and sum of neonatal weights.

#### Delivery and surgical methods

Vaginal operation, delivery assistance, and cesarean section were all performed by midwives and doctors with midwifery qualifications and surgical qualifications.

### Statistical analysis

SPSS 26.0 and R 4.2.2 software were used for analysis. Continuous variable data that were not normally distributed were described by medians and quartiles, and the frequency of categorical variables were expressed as percentages [n ($\%$)]. The Mann-Whitney U test was used for differences in continuous variables. In contrast, the chi-square test was used for differences in count data. In the training cohort, LASSO regression screening was used to identify predictor variables. The predictor variables found in the LASSO regression were further included in the stepwise multifactorial logistic regression analysis. Multivariate logistic regression analysis was used to identify independent risk factors. Odds ratios and their corresponding 95$\%$ confidence intervals were then calculated for each independent risk factor. P values less than 0.05 were considered statistically significant at the 95$\%$ confidence level. A nomogram was established based on the results of multiple logistic regression analysis. Nomogram performance was quantified using the receiver operating characteristic (ROC) curve, Hosmer- Lemeshow test and decision curve analysis (DCA).

## Results

### Patient characteristics

**Fig. 1:**
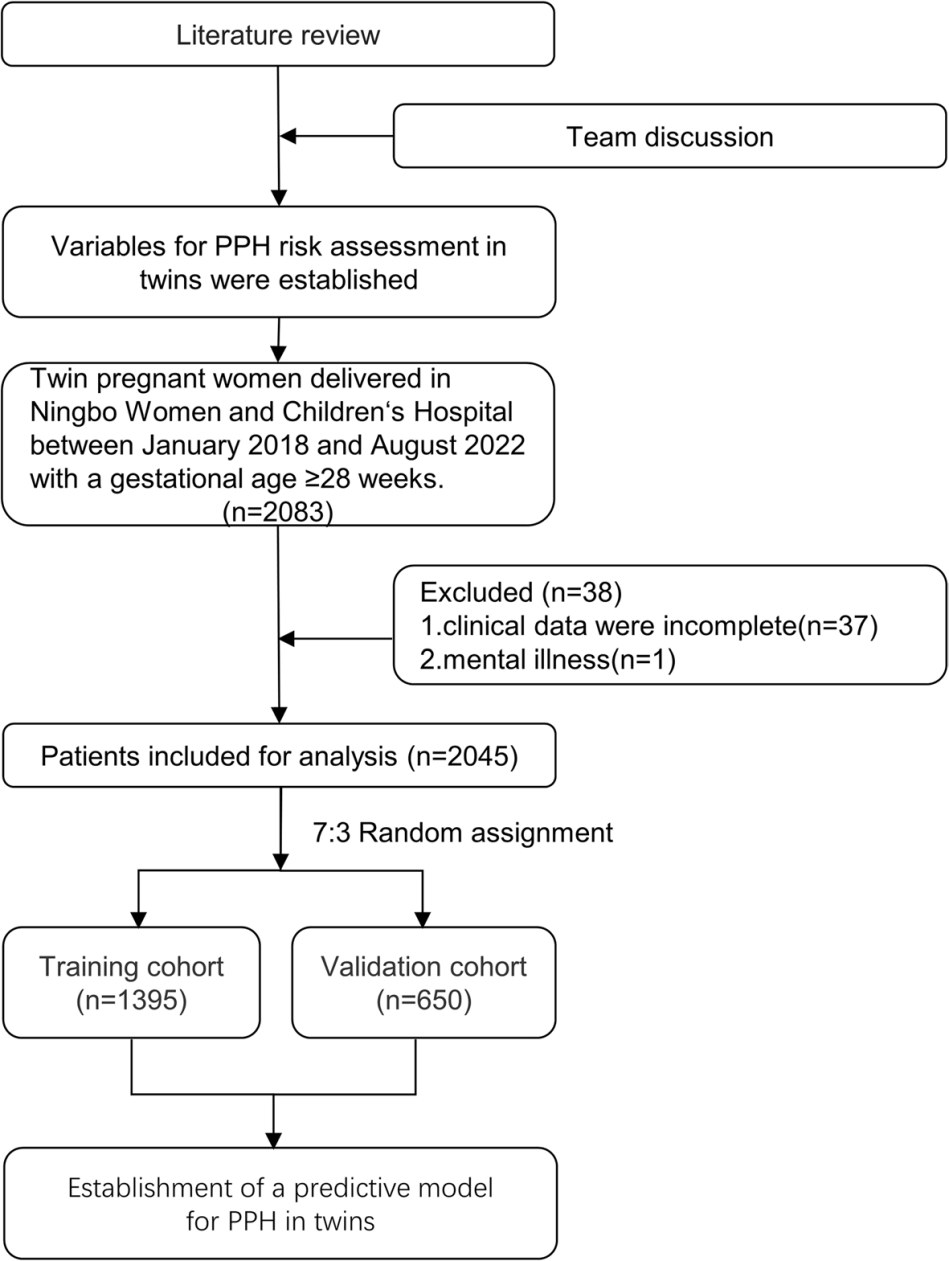
Flowchart of participants in the study

Finally, 2,045 cases met the inclusion criteria, with an inclusion rate of 98.2$\%$ ($n=2045/2083$). Patients included in the study were randomly divided into a training cohort ($n=1395$) and a validation cohort ($n=650$) at a ratio of 7:3 (Fig. 1). There were no statistically significant differences between the training and validation cohorts in general patient data, prenatal factors, previous factors, or delivery factors (*P* > 0.05), and the training and validation cohorts had good homogeneity (Additional file 1).

### Regression analysis and risk factors for PPH

In the training cohort sample, LASSO regression analysis identified 12 predictor variables with nonzero coefficients from 27 variables. Vertical lines were plotted at the minimum value of $\lambda$ ($\lambda$ = 0.003) and the 1SE of the minimum value ($\lambda$= 0.022), and the 12 nonzero coefficient predictor variables were screened at log(0.022) = -3.806 when the LASSO regression model was most appropriate (Additional file 2). Twelve predictor variables screened by LASSO regression analysis were used as independent variables, and multifactorial logistic results showed that maternal age, assisted reproduction, platelet count, fibrinogen level, albumin level, hypertensive disorders of pregnancy, placenta previa, number of previous cesarean deliveries, number of previous intrauterine manipulations and the sum of neonatal weights were independent risk factors for PPH in twins ($P<0.05$) (Table 1).

**Table 1:** Multifactorial logistic regression analysis of the risk of PPH in twin pregnancies

Intercept and Variable	B	SE	Z	P	OR	95$\%$CI
intercept	-3.375	1.241	-2.719	0.007	0.034	(0.003$\sim$0.385)
maternal age	0.054	0.021	2.609	0.009	1.056	(1.013$\sim$1.099)
ART						
No					1	
Yeas	1.088	0.217	5.005	$<0.001$	2.969	(1.957$\sim$4.596)
PLT	-0.004	0.002	-2.142	0.032	0.996	(0.993$\sim$1.000)
FIB	-0.003	0.001	-3.084	0.002	0.997	(0.995$\sim$0.999)
albumin level	-0.065	0.025	-2.574	0.010	0.937	(0.892$\sim$0.985)
HDP						
No					1	
gestational hypertension	0.444	0.338	1.312	0.190	1.559	(0.777$\sim$2.950)
chronic hypertension	0.442	1.272	0.348	0.728	1.556	(0.068$\sim$16.333)
eclampsia, preeclampsia, superimposed preeclampsia	1.642	0.218	7.518	$<0.001$	5.166	(3.368$\sim$7.939)
placenta praevia						
No					1	
Yes, non-anterior placenta	2.536	0.472	5.371	< 0.001	12.635	(5.068$\sim$32.782)
Yes, anterior placenta	2.373	1.294	1.833	0.067	10.732	(0.887$\sim$249.999)
number of previous cesarean deliveries						
0					1	
1	0.763	0.357	2.134	0.033	2.144	(1.034$\sim$4.232)
$\ge 2$	3.588	1.219	2.944	0.003	36.154	(4.059$\sim$786.363)
number of previous intrauterine manipulation						
0					1	
$\ge 1$	1.223	0.173	7.062	$<0.001$	3.396	(2.421$\sim$4.777)
a sum of neonatal weigh	0.001	0.000	4.602	$<0.001$	1.001	(1.000$\sim$1.001)

*Abbreviations:* *ART* assisted reproduction, *PLT* platelet count, *FIB* fibrinogen level, *HDP* hypertensive disorders of pregnancy, *OR* odds ratio, *CI* confidence interval

### Model construction and validation

A nomogram was drawn based on the predictor variables (Additional file 3). To facilitate calculations, each independent risk factor affecting PPH was quantified, and each predictor variable’s scores were added together to create the overall score (Table 2). with each predictor given a score according to the characteristics of each patient; the total number of scores was then calculated to obtain the risk of PPH.

**Table 2:** The scores of each Variable of PPH in twin pregnancies

Variable	Value	Score
maternal age	0.054	1.638 × maternal age -24.572
ART		
No	0	0
Yeas	1.088	37.944
PLT	-0.004	-0.118 × PLT + 59.271
FIB	-0.003	-0.0956 × FIB + 81.250
albumin level	-0.065	-1.865 × albumin level + 89.503
HDP		
No	0	0
gestational hypertension	0.444	12.263
chronic hypertension	0.442	13.381
eclampsia, preeclampsia, superimposed preeclampsia	1.642	49.525
number of previous cesarean delivery		
0	0	0
1	0.763	23.700
$\ge 2$	3.588	70.898
number of previous intrauterine manipulation		
0	0	0
$\ge 1$	1.223	36.985
a sum of neonatal weigh	0.001	0.0167 × sum of neonatal weigh-25.000

*Abbreviations:* *ART* assisted reproduction, *PLT* platelet count, *FIB* fibrinogen level, *HDP* hypertensive disorders of pregnancy, *OR* odds ratio, *CI* confidence interval

**Fig. 2:**
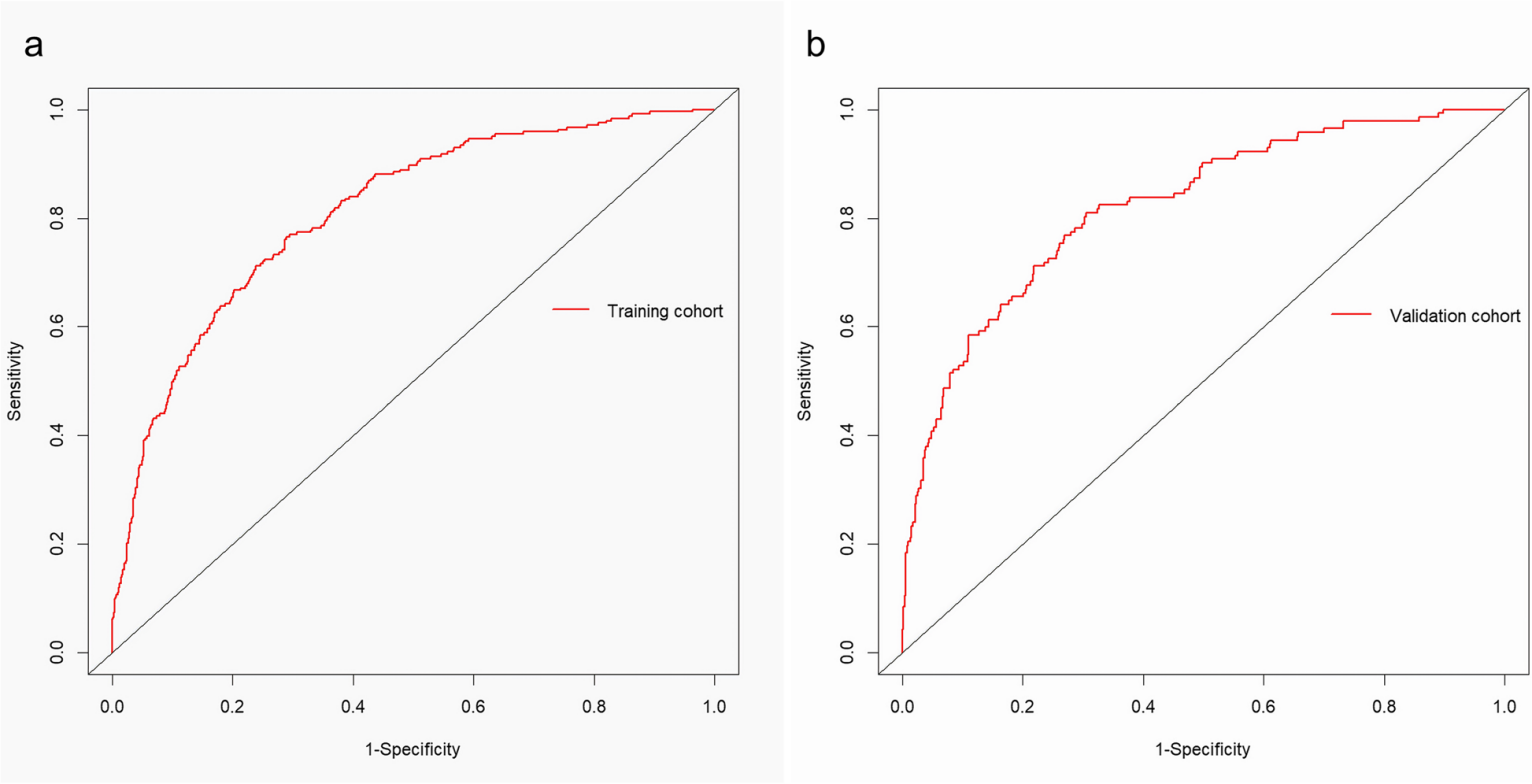
ROC curve of the PPH risk prediction model in twin pregnancies

The discriminatory power of the predictive model was analysed using ROC curve (Fig. 2). The area under the curve (AUC) value for the training cohort was 0.810 [95$\%$ CI (0.781, 0.839)], with a sensitivity of 76.54$\%$ [95$\%$ CI (0.707, 0.817)]; specificity of 71.01$\%$ [95$\%$ CI (0.683, 0.736)]; PPV and NPV of 0.358 and 0.935, respectively; and a cut-off value of 0.161, with a Youden’s index of 0.476. The AUC value for the validation cohort was 0.821 [95$\%$ CI (0.781, 0.860)], with a sensitivity of 80.9$\%$ [95$\%$ CI (0.736, 0.871)]; specificity of 69.49$\%$ [95$\%$ CI (0.653, 0.735)]; PPV and NPV of 0.426 and 0.929, respectively; and a cut-off value of 0.191, with a Youden’s index of 0.476. The cut-off value was 0.191, and Youden’s index was 0.505. That shows the prediction model had a good calibration.

**Fig. 3:**
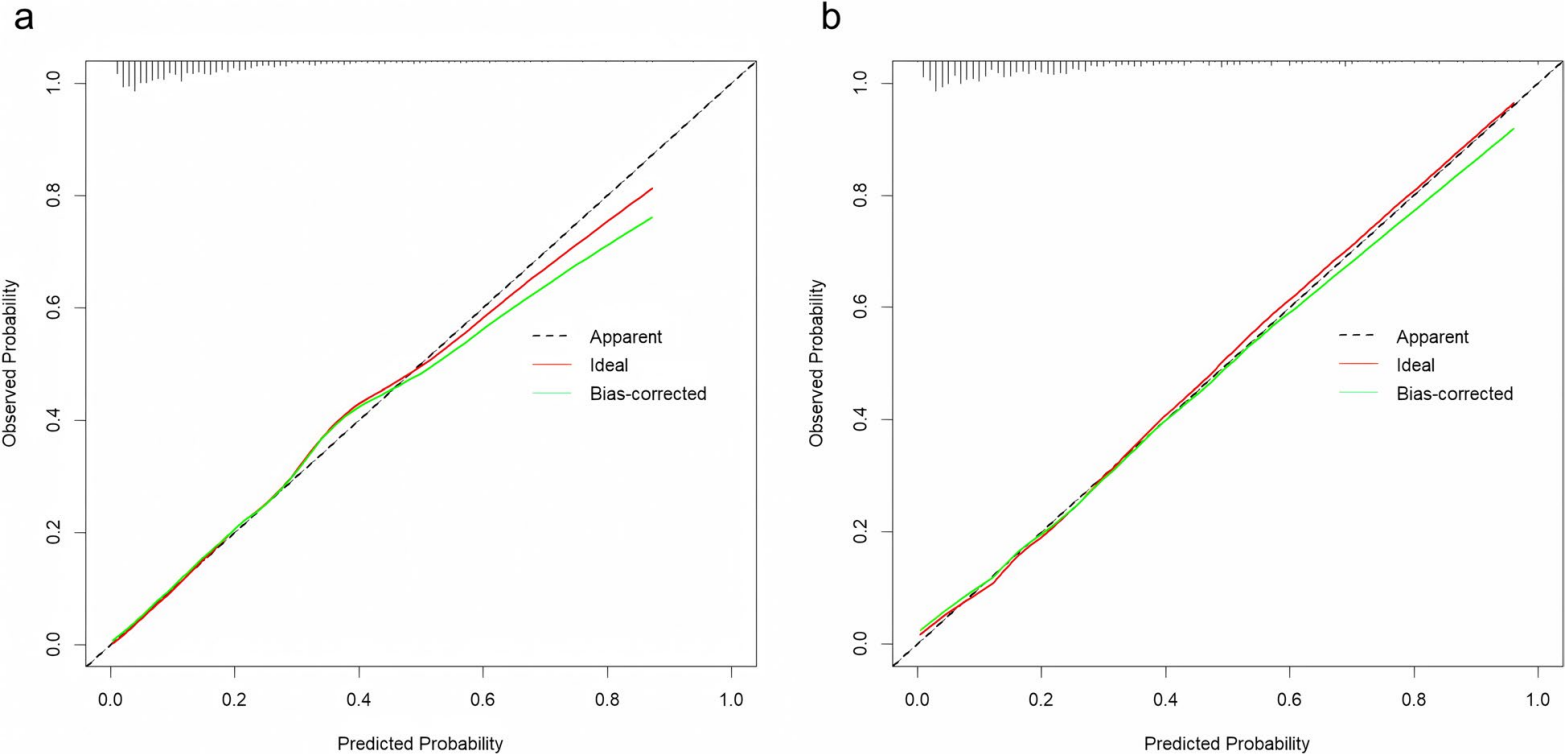
Calibration curve for the predictive model for PPH in twin pregnancies

The model was internally validated with 1000 bootstrap samples. The Hosmer-Lemeshow goodness-of-fit test showed a good fit with *X*$^{2}$ = 0.885, (*P*= 0.642) for the training cohort, and *X*$^{2}$ = 0.332, (*P*= 0.846) for the testing cohort, indicating that the model predicted probabilities were generally consistent with the actual probabilities, and the prediction model had good correction ability (Fig. 3).

### Clinical application value of the model

The clinical validity of the prediction model was assessed by decision curve analysis. The decision curve shows that the threshold probability of adverse outcomes of PPH in pregnant women is in the range of 3$\%$-96$\%$, and if treatment measures are taken, the treatment of patients at such a time would have a net benefit (Additional file 4).

### Web-based dynamic nomogram

Considering the complexity of the steps in the actual clinical application of the nomogram, this study established a web-based dynamic nomogram: https://stchcxycmx.shinyapps.io/hpprisk/. It can be added to a computer or phone for easy and convenient use. Additional file 5 shows a screenshot of the web page for the dynamic nomogram model.

## Discussion

Twin pregnancy is a significant risk factor for PPH, and doctors can be better prepared to handle PPH and avoid severe morbidity if the high-risk individual is detected early and adequately [7].

There have been a few studies using a prediction model for PPH in twin pregnancies at home and abroad. Statistical analysis was performed by I Yuting Zhu et al. [8] on 734 twin pregnancies at term. The model’s AUC was 0.706 [95$\%$ CI (0.645, 0.768)], while the validation group’s AUC was 0.726 [95$\%$ CI (0.617, 0.835)], with a sensitivity of 51.8$\%$ and a specificity of 78.7$\%$. The disadvantage of this model is that the accuracy of the nomogram’s prediction was low, and the surgeon or midwife determined the amount of postpartum hemorrhage based on personal experience, which may lead to some errors. Tang Hui et al. [9] quantified the included risk factors in the form of “variable assignment” in 763 twin cases, weighted multiple risk factors, and divided patients into three groups, using the total score to predict the rate of PPH. The limitation of this study is the small sample size. Kartik K Venkatesh et al. [10] compared prediction models for PPH in four types of twins using general data from 152,279 pregnant women obtained from the American Safe Labor Alliance; however, specific crucial clinical characteristics were missing (e.g., platelet count, thromboprophylaxis medication, placenta praevia, macrosomia, and uterine fibroids were not included in the dataset). In summary, there is still a need to develop predictive models that are widely recognized and used in clinical practice.

In this study, placenta previa was the factor that had the largest effect on PPH in twin pregnancies. It is a significant factor in severe PPH and even hysterectomy in pregnant women [11], and in a meta-analysis of a study, placenta previa accounted for 32.3$\%$ of patients with severe PPH, and placenta implantation accounted for 33.8$\%$ [12]. The placenta attaches to the anterior wall of the uterus, which can significantly impact the amount of postpartum bleeding. For example, placenta previa of the anterior wall necessitates a cesarean section, which requires cutting a hole through the placenta to remove the foetus; this procedure leaves the placenta incompletely dissected, which increases the amount of postpartum bleeding. Compared to singleton pregnancies, twin pregnancies are more likely to experience placenta previa and placenta accreta [13].

Patients undergo hysteroscopy to analyse the endometrium for assisted reproduction; this increases the frequency of hysteroscopic surgeries, which can easily cause Asherman syndrome, increasing the risk of decidua dysplasia in subsequent pregnancies along with placenta previa, placenta accreta, and placenta percreta. Assisted reproduction has been identified as a high-risk factor for PPH in twin pregnancies [14, 15]. More research is needed to determine whether ovulation induction drugs affect PPH [16]. Long-term psychological pressure, combined with family, social, and economic pressure, causes pregnant women who require assisted reproduction to experience a high amount of stress, which can lead to neurological disorders, reduce posterior pituitary hormone secretion, and increase the risk of PPH [17].

According to a prospective study based on a national twin delivery population, the risk of severe PPH increases linearly with the sum of the birth weights of the twins [18]. This is because an increase in foetal weight causes the uterus’ myofibers to stretch, which results in uterine atony after delivery. It also affects the proportion of placental area in women with sizeable foetal weight, which causes a blood sinus to open when the placenta is detached. The total foetal weight is also more likely to result in extended labour and soft birth canal lacerations, raising the risk of PPH during vaginal delivery.

Surgeries of the uterine cavity include artificial abortion, diagnostic curettage, and hysteroscopic surgery, which results in endometritis and intrauterine adhesions. In particular, the history of the division of endometrial synechiae is a risk factor for placenta previa and placenta percreta caused by recurrent pregnancy, with incidence rates of placenta praevia, placenta percreta, and PPH reported to be 8.11$\%$, 17.57$\%$, and 21.62$\%$, respectively [19]. History of cesarean section is a high-risk factor for PPH; in pregnant women who have had three cesarean sections, the frequency of placenta previa is as high as 14.2$\%$. However, placental implantation occurs in 4$\%$, 14$\%$, 23$\%$, 35$\%$, and 50$\%$ of women who have had 0, 1, 2, 3, and 4 cesarean births, respectively [12].

Twin pregnancies are particularly susceptible to HDP, which increases the risk of PPH [20]. HDP causes spasms of systemic small blood vessels, and abnormal blood flow to the placenta, leading to uterine myxoedema; in addition, the use of antihypertensive drugs in the clinic may reduce the excitability of uterine muscle fibres, leading to compromised uterine contractile function and increased PPH risk.

## Conclusion

Our nomogram risk prediction method is both practical and straightforward to use, and it has the potential to be employed in clinical practice to predict the occurrence of PPH in twin pregnancies. Compared to other studies, our PPH prediction model involved a larger sample size and has a higher prediction accuracy. Furthermore, it is provided in the form of an easy-to-use online calculator, which can be saved to a computer or mobile phone and used to determine the estimated probability of postpartum hemorrhage by accessing the web-based application and entering data of the general state of pregnant women before birth directly. The current study has some limitations, such as data from a single site and only internal testing of the model; our model needs to be validated in more extensive and diverse populations.

### Supplementary Information

**Additional file 1.**

**Additional file 2.**

**Additional file 3.**

**Additional file 4.**

**Additional file 5.**

## Data Availability

The datasets generated during the current study are not publicly available because they contain personal information about patients, but can be obtained from the corresponding author as needed.

## Abbreviations

PPH: Postpartum hemorrhage
ART: Assisted reproduction
BMI: Body mass index
PLT: Platelet count
FIB: Fibrinogen level
GDM: Gestational diabetes mellitus
ICP: Intrahepatic cholestasis of pregnancy
HDP: Hypertensive disorders of pregnancy
PROM: Prelabour rupture of membranes
ROC: Receiver operating characteristic
AUC: Area under the curve
DCA: Decision curve analysis
CI: Confidence intervals
PPV: Positive predictive value
NPV: Negative predictive value
