# Supplementary material for: Establishment of a predictive model for postpartum hemorrhage in twins: a retrospective study
Source: BMC Pregnancy Childbirth. 2023 Sep 7;23:644. doi: 10.1186/s12884-023-05933-7 (PMC10486133; doi:10.1186/s12884-023-05933-7)
Supplement: Supplementary file 2 — Additional file 2. [file 12884_2023_5933_MOESM2_ESM.docx]

Additional file 2 --- LASSO regression analysis and risk factors for PPH in twin pregnancies


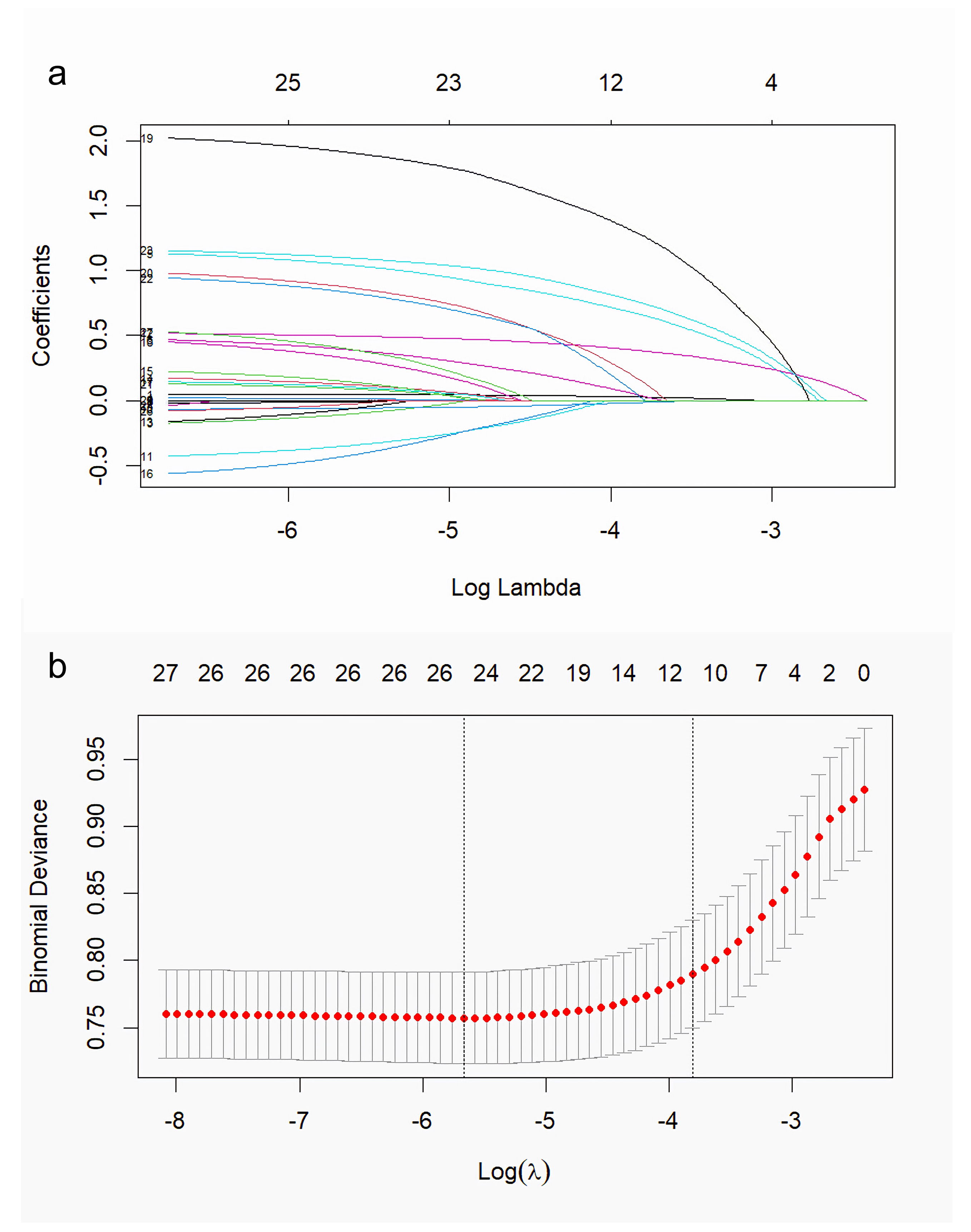


a: LASSO regression model to filter predictors; b: shows the process of selecting the most suitable λ in the LASSO model through 10-fold cross-validation. LASSO coefficient profiles of the 12 features, including maternal age, assisted reproduction, type of chorionic membrane, platelet count, fibrinogen level, albumin level, hypertensive disorders of pregnancy, placenta praevia, number of previous cesarean deliveries, number of previous intrauterine manipulation, history of previous PPH and the sum of neonatal weight.
