# Supplementary material for: Establishment of a predictive model for postpartum hemorrhage in twins: a retrospective study
Source: BMC Pregnancy Childbirth. 2023 Sep 7;23:644. doi: 10.1186/s12884-023-05933-7 (PMC10486133; doi:10.1186/s12884-023-05933-7)
Supplement: Supplementary file 3 — Additional file 3. [file 12884_2023_5933_MOESM3_ESM.docx]

Additional file 3 --- Predictive nomogram model of PPH risk in twin pregnancies


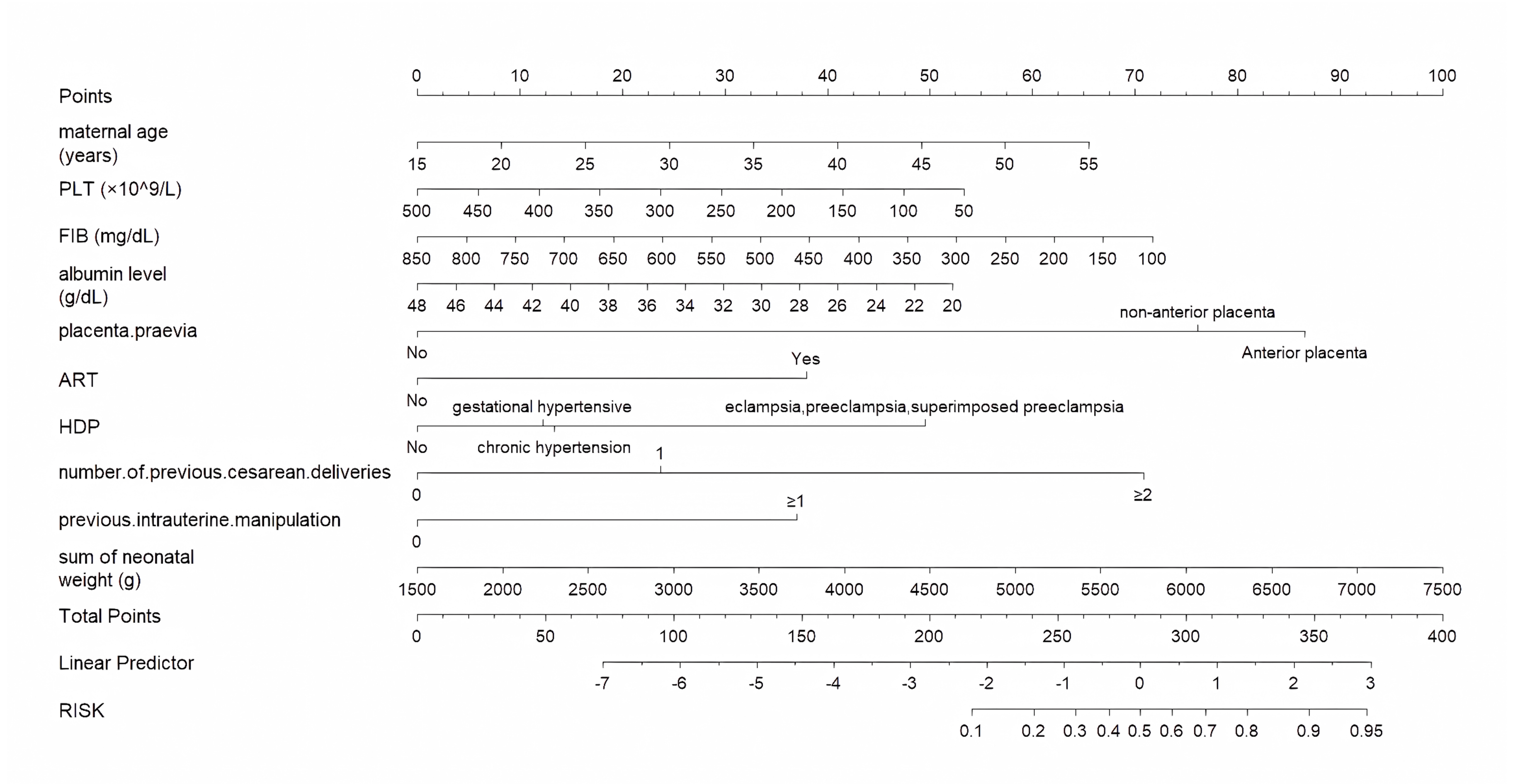


By drawing a straight upward line to the Points axis, each variable corresponds to a specific point. By drawing a straight line down to the probability axis after totaling the points on the Total points axis, the sum shows the risk of PPH in twins.
