# Supplementary material for: Establishment of a predictive model for postpartum hemorrhage in twins: a retrospective study
Source: BMC Pregnancy Childbirth. 2023 Sep 7;23:644. doi: 10.1186/s12884-023-05933-7 (PMC10486133; doi:10.1186/s12884-023-05933-7)
Supplement: Supplementary file 4 — Additional file 4. [file 12884_2023_5933_MOESM4_ESM.docx]

Additional file 4 --- DCA curve of the predictive model for PPH in twin pregnancies


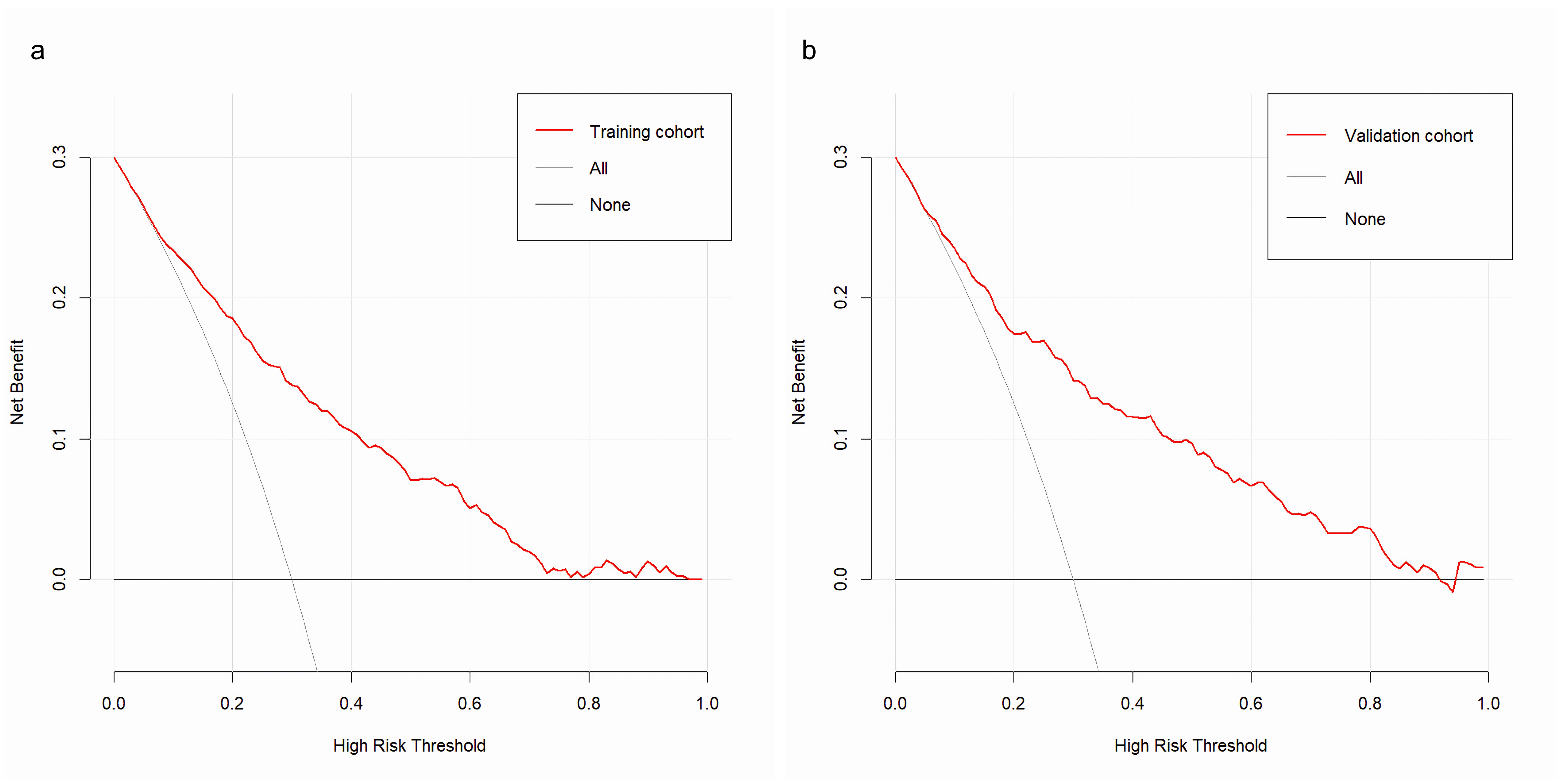


DCA of the nomogram. a: training cohort b: validation cohort
