# Supplementary material for: Establishment of a predictive model for postpartum hemorrhage in twins: a retrospective study
Source: BMC Pregnancy Childbirth. 2023 Sep 7;23:644. doi: 10.1186/s12884-023-05933-7 (PMC10486133; doi:10.1186/s12884-023-05933-7)
Supplement: Supplementary file 5 — Additional file 5. [file 12884_2023_5933_MOESM5_ESM.docx]

Additional file 5 --- Web version of the dynamic nomogram for PPH in twin pregnancies


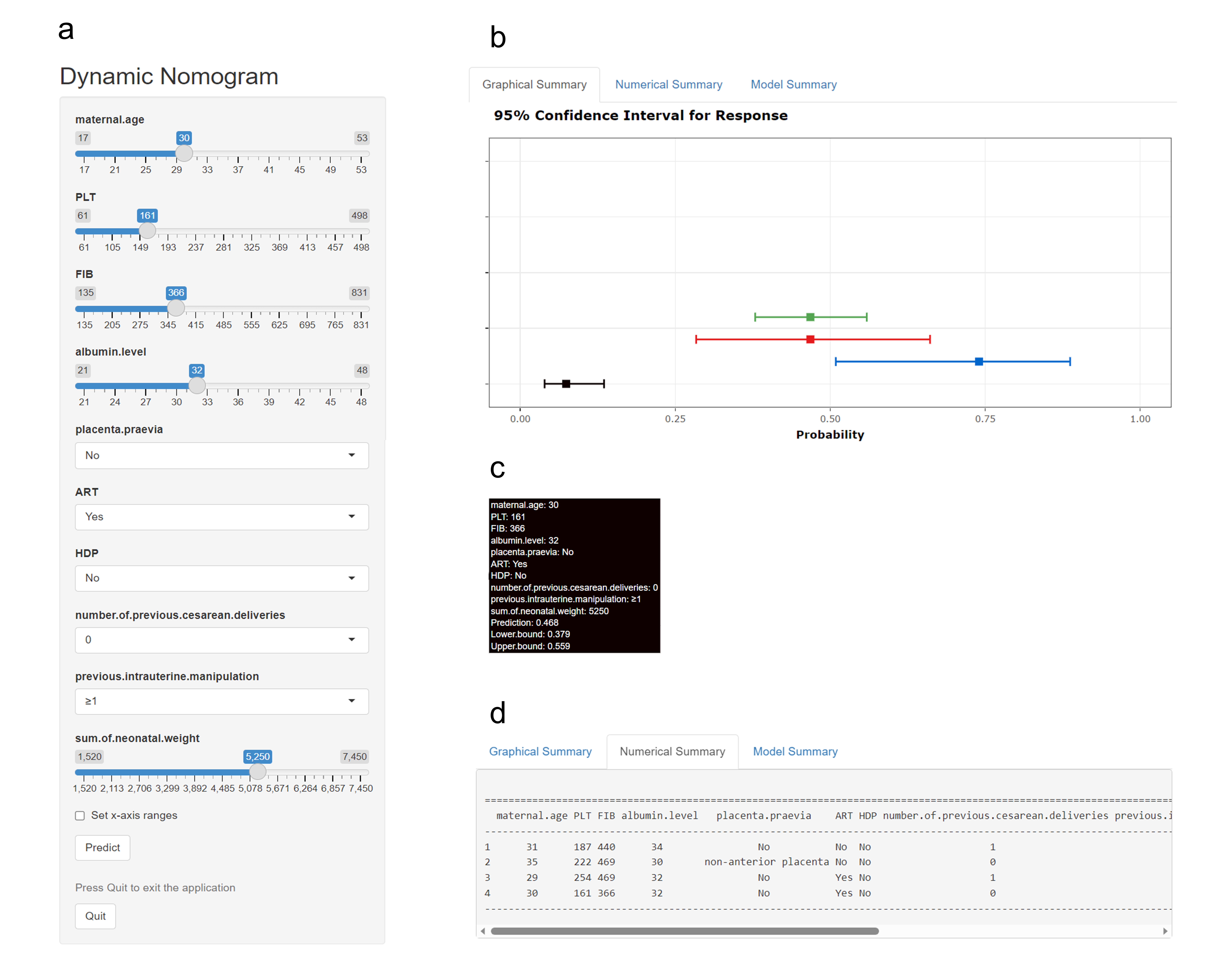


Web-based dynamic nomogram for PPH in twins. By entering the specific information of the women with twin pregnancie in the web-online tool, we could obtain the participant’s PPH probability. a. Entering Interface: You can enter the specific information of the participant in this interface. b. Graphical Summary: The PPH probability and 95% confidence interval of participants are depicted in this interface. c. Graphical Summary: Summary of the specific information, the PPH probability and 95% confidence interval. d. Numerical Summary: PPH probability and 95% confidence interval are shown in this interface.
